# Comparison of Venous Thromboembolism after Total Hip Arthroplasty between Ankylosing Spondylitis and Osteoarthritis

**DOI:** 10.1155/2014/712895

**Published:** 2014-06-04

**Authors:** Dongquan Shi, Xingquan Xu, Kai Song, Zhihong Xu, Jin Dai, Dongyang Chen, Qing Jiang

**Affiliations:** ^1^The Center of Diagnosis and Treatment for Joint Disease, Drum Tower Hospital Affiliated to Medical School of Nanjing University, Zhongshan Road 321, Nanjing, Jiangsu 210008, China; ^2^Laboratory for Bone and Joint Diseases, Model Animal Research Center, Nanjing University, Nanjing, Jiangsu 210061, China

## Abstract

*Objective.* Ankylosing spondylitis (AS), an inflammatory rheumatic disease, will gradually lead to severe hip joint dysfunction. Total hip arthroplasty is a useful method to improve patients' quality of life. The aim of this study was to compare the incidence and risk factors of deep vein thrombosis (DVT) between AS and hip osteoarthritis. *Methods.* In a retrospective study, a total of 149 subjects who underwent cementless THA were studied. Clinical data, biochemical data, and surgery-related data were measured between AS and OA groups. *Results.* The incidence of DVT in AS group was lower than that of OA group, although no significant difference was detected (*P* = 0.89). The patients of AS group were much younger (*P* < 0.0001) and thinner (*P* = 0.018) compared with those of OA group. AS patients had higher ejection fraction (EF) (*P* = 0.016), higher platelet counts (*P* < 0.0001), and lower hypertension rate (*P* = 0.0004). The values of APTT, PT, and INR in AS patients were higher than those in OA patients (all *P* < 0.0001). The values of D-dimer and APTT were both significantly higher in DVT subjects than those in non-DVT subjects. *Conclusion.* AS patients potentially had a lower incidence of DVT compared with OA patients.

## 1. Introduction

Venous thromboembolism (VTE), which encompasses deep vein thrombosis (DVT) and pulmonary embolism (PE), is a potentially life-threatening complication [1] and is associated with significant morbidity and mortality. As operation of the century [2], total hip arthroplasty (THA) makes people walk easier. However, VTE is the most severe complication after THA.

Immune system and coagulation system are linked. Many molecular components are important for both systems [3–5]. Comparative studies indicated that coagulation and innate immunity have a common evolutionary origin [6, 7]. Some of the central features of the hypercoagulability induced by inflammation are cytokine induction of tissue factor (TF) expression, endothelial dysfunction, inhibition of the protein C system and inhibition of fibrinolysis (increased plasminogen activator inhibitor 1 levels) [3–5] and platelets, microparticles (MP), neutrophils, thrombin and protease-activated receptors, fibrinogen, *α*1-antitrypsin, heparin proteoglycans, and the contact system (Factor XII and the kallikrein-kinin system). Ankylosing spondylitis (AS) is a chronic inflammatory disease, with an increased cardiovascular risk [8, 9]. Wendling and Racadot found serum tissue factor levels which are correlated with inflammation are elevated in AS compared to controls [10]. Thrombin generation test has been used to evaluate plasma-based hypercoagulability [11–13]. And thrombin plays a critical role in regulating coagulation and fibrin clot formation [14]. Thrombin also increases the secretion of inflammatory cytokine and angiogenic growth factor [15], playing a role in the process of inflammation [16].

The aim of this study was to evaluate and compare thrombotic risk after THA between patients with AS and those with noninflammatory arthritis, osteoarthritis (OA), to clarify whether AS represents a risk factor to VTE following THA.

## 2. Methods

### 2.1. Participants

From January 2007 to April 2010, 908 subjects of Nanjing DVT study (NJDVTS) received venography after hip or knee surgery. A total of 149 subjects (72 women and 77 men) who underwent cementless THA using lateral approach performed by one skilled orthopedic surgeon were studied. AS with HLA-B27 positive was diagnosed by two senior doctors. Subjects in control group with primary or secondary osteoarthritis were included. All the subjects were enrolled consecutively at Center of Diagnosis and Treatment for Joint Disease, Drum Tower Hospital, affiliated to Medical School of Nanjing University. All subjects included in the study were Han Chinese living in and around Nanjing. No subjects dropped out during the study. The study was approved by the Ethical Committee of Medical School of Nanjing University, and informed consent was obtained from the subjects included.

### 2.2. Clinical Management and Assessment of DVT

All the patients received 0.3 mL (38 International Factor Xa Inhibitory Units per kilogram of body weight) of low-molecular-weight heparin from the same company subcutaneously once daily. The dosage level used as prophylaxis is considered to be moderate. The first injection was given 12 to 16 hours after surgery if there was no clinically evident bleeding. Prophylaxis was continued until venography was performed. All the patients were examined by contrast venography (CV) 3–5 days after operation and diagnosed by 3 experienced doctors. DVT was diagnosed according to the Robinov group's criterion [17]. PE was diagnosed by CT scan for pulmonary artery angiogram. If VTE was detected, conventional thrombolysis treatment was to be started. If not, patients would not receive any further anticoagulation treatment.

### 2.3. Measurements

Age, sex, DVT related history, diabetes mellitus (DM), hypertension, cancer, hormone therapy, and smoking history were recorded. We measured clinical and biochemical data (weight, height, coagulation related data, fibrinogen, RBC, PLT, and D-dimmer) and surgery-related data (duration of surgery, tourniquet time, EF, Hb, and symptoms of DVT). Visual analogue scale (VAS) was described after operation.

### 2.4. Statistical Analysis

For the comparison of independent factors between the two groups, *t*-test was used. We assessed incidence of DVT between two groups by the *χ* ^2^ test. *P* < 0.05 was considered statistically significant. All the statistical analyses were performed using SPSS 15.0 system software.

## 3. Results

### 3.1. Demographic Characteristics

Patient demographic characteristics were shown in Table 1. The mean age of AS group (41.8 years, range of 17–73 years) was significantly younger than OA group (63.1 years, range of 20–87 years). BMI of OA group (24.96 ± 8.54) was statistically higher than AS group (21.83 ± 3.69). The proportion of female subjects in AS (8/54 = 14.8%) was significantly smaller than OA group (64/95 = 67.4%) (*P* < 0.0001). The values of ejection fraction (EF) of AS (62.04 ± 2.73%) were significantly higher than OA patients (60.59 ± 2.66%) (*P* = 0.016). Erythrocyte sedimentation rate (ESR) in AS group and OA group was 33.17 ± 11.91 and 28.86 ± 11.6, respectively. Platelet counts in AS group (200.61 ± 69.2) were more than those in OA group (146.26 ± 44.1) (*P* < 0.0001). In contrast, incidence of hypertension in AS (4/54 = 7.4%) was lower than that in OA group (35/95 = 36.8%). Incidence of stroke history was more frequent in OA group.

### 3.2. Incidence of DVT

CV confirmed DVT was detected in 8 patients in the AS group (14.8%) and 17 patients in the OA group (17.9%). The *P* value was larger than 0.05. No proximal DVT in AS group was detected, while there was one in OA group (1.1%) (Table 2).

Coagulation related data included activated partial thromboplastin time (APTT), partial thromboplastin time (PT), international normalized ratio (INR), and thrombin time (TT).

The values of APTT, PT, and INR in AS patients (31.76 ± 7, 12.14 ± 1.86, and 1.06 ± 0.15) were higher than those in OA patients (25.53 ± 3.41, 11.12 ± 0.82, and 0.97 ± 0.07). Significant differences of APTT, PT, and INR between AS and OA groups were detected preoperatively (all *P* values < 0.001). When stratified by patients with DVT or not, we found APTT in patients with DVT (38.9 ± 13.86) was significantly higher than that with non-DVT (29.89 ± 6.12) (*P* = 0.04). However, no statistical difference was detected in other stratification analyses postoperatively. Detailed data was shown in Table 3.

### 3.3. Pain and DVT

VAS scores were evaluated for all the patients. VAS scores were 3.19 (0–6) and 3.16 (0–7) for DVT and non-DVT groups, respectively. No significant difference was detected between DVT and non-DVT groups (*P* = 0.91). When stratified by two conditions, in AS group, VAS score of DVT subjects (1.33, from 0 to 2) was lower than that of non-DVT subjects (3.37, from 0 to 7). In OA group, VAS score of DVT subjects (3.12, from 1 to 6) was lower than that of non-DVT subjects (3.5, from 1 to 5). However, no significant difference was detected between VAS score and incidence of DVT.

### 3.4. D-Dimer and DVT (Table 4)

D-dimer was tested for all the patients. Concentration of D-dimer in DVT subjects (3.88 ± 5.40) was significantly higher than that in non-DVT subjects (2.12 ± 1.97). Significant difference was detected between DVT and non-DVT groups (*P* = 0.01). Stratification was performed for AS and OA groups. Statistical difference was detected between DVT (2.54 ± 0.45) and non-DVT subjects (0.76 ± 0.92) in AS group (*P* = 0.01). In OA group, D-dimer of DVT subjects (4.05 ± 5.72) was higher than that of non-DVT subjects (2.61 ± 2.02). However, no statistical difference was detected. (*P* = 0.09).

### 3.5. D-Dimer and DVT (Table 5)

D-dimer was tested for all the patients. Concentration of D-dimer in DVT subjects (3.88 ± 5.40) was significantly higher than that in non-DVT subjects (2.12 ± 1.97). Significant difference was detected between DVT and non-DVT groups (*P* = 0.01). Stratification was performed for AS and OA groups. Statistical difference was detected between DVT (2.54 ± 0.45) and non-DVT subjects (0.76 ± 0.92) in AS group (*P* = 0.01). In OA group, D-dimer of DVT subjects (4.05 ± 5.72) was higher than that of non-DVT subjects (2.61 ± 2.02). However, no statistical difference was detected. (*P* = 0.09).

## 4. Discussion

To our knowledge, our study was the first one to evaluate and compare the incidence of DVT between AS and OA patients after THA. THA is a useful strategy to improve the life quality of late-stage AS patients as they often suffered from severe hip joint disability. A common and severe complication of THA is VTE which is a multifactorial disease caused by both individual and surgery-related factors. Despite effective prophylaxis, subclinical VTE develops soon after surgery in 15 to 20% of patients who undergo THA. However, the incidence of patients with AS after THA is unknown. In this study, we conducted a retrospective study to compare the incidence and risk factors between AS and OA after THA. Our results showed a decreased risk of developing VTE for AS patients compared with OA patients. Our study gives insight into the relative importance of clinical risk factors associated with VTE after THA.

Our study showed that the incidence of DVT in AS subjects was lower compared with OA patients, although no significant difference was found. The exact reasons were not clear, as the factors that can influence the incidence of DVT are often complex. The connection between AS and VTE is not yet clearly understood. Several reports showed active systemic inflammation may play a role in hypercoagulability, as endothelial dysfunction and inhibition of fibrinolysis which is related to the process of thrombosis can be induced by cytokines such as TNF and IL-1. [18–20]. There is also a report that showed that higher platelet count was associated with an elevated risk of VTE [21]. In the present study, higher platelet count was found in AS subjects compared with OA patients (Table 1). Patients with AS might have other risk factors of VTE such as difficult surgical procedures, physical inactivity, and cardiovascular comorbidities compared to OA patients. In our study, longer duration of surgery, higher platelet count, and active systemic inflammation in patients with AS were found compared to OA patients which all indicated a higher potential incidence of DVT. However, in our study, patients with AS were much younger and thinner than those with OA. It has been reported that increased age and high BMI are strong and prevalent risk factors for onset of DVT after THA [22]. Ageing may also be associated with an increased prevalence of other risk factors. For example, elder subjects have lower value of EF, which may promote thrombosis. Also, in the elder subjects, women accounted for the higher proportion. More female patients were found in OA subjects than AS subjects in our study (Table 1). There are also fewer patients who suffered from hypertension in AS subjects. These features might make AS patients less susceptible to DVT.

The activated partial thromboplastin time (APTT) is an indicator which can measure the efficacy of both the common coagulation and “intrinsic” (now referred to as the contact activation pathway) pathways. APTT is often used combined with the prothrombin time (PT), as PT measures the extrinsic pathway. Sorensen and Ingerslev reported that a shortened APTT indicated an increased risk of VTE [23]. INR was used as a monitor to reflect the treatment effects of heparin. In the present study, the values of APTT, PT, and INR were all higher in AS subjects than those of OA patients (Table 1). This was consistent with literatures as the incidence trend of VTE in AS group was lower than OA group in our study. In fact, in AS subjects, the patients who had DVT after surgery showed much higher value of APTT than patients without DVT (*P* = 0.036). So, our results indicated APTT can be used to predict the risk of DVT in patients with AS.

Based on the clinical experiences, postoperative pain can affect the outcome and other complications of THA. The severe postoperative pain leads to inactive rehabilitation exercise and extended hospital stays and can result in overall low satisfaction and potentially greater cost [24]. Additionally, postoperative pain can reduce range of motion and frequencies of ankle pump [25, 26]. These all mean that higher VAS score indicates a higher risk of DVT after surgery. However, in our study, patients with DVT felt shorter and lighter pain than patients without DVT in AS group. This phenomenon may come from the small number of DVT patients in AS. Higher level clinical trials are needed to illustrate the association between incidence of DVT and pain.

D-dimer is a marker of endogenous fibrinolysis and should therefore be detected in patients with deep vein thrombosis [27]. D-dimer assays with high sensitivity and low specificity are fast, accurate, and readily available [28, 29]. Negative results with well-validated clinical manifestation can exclude VTE. However, many conditions like renal insufficiency, hematoma, bleeding, and atrial fibrillation can cause a high value of D-dimer [30–33]. In our study, higher value of D-dimer was detected in subjects with VTE compared to that with non-DVT. No significant difference was found between AS and OA group before operation. In AS group, significant higher value of D-dimer was detected in DVT group. D-dimer for predicting DVT might be more valuable in autoimmune system disease than in degenerative disease. Studies with larger number of subjects would be required to confirm the results and explore the mechanism.

In conclusion, our results showed a decreased risk of developing VTE for AS patients compared with OA patients.

## Figures and Tables

**Table 1:** Characteristic data of the subjects.

	AS	OA	*P*
Number of hips	54	95	
Number of patients	48	95	
Age (years old)	41.8 (17–73)	63.1 (20–87)	<0.0001
Sex	54	95	<0.0001
Male	46	31	
Female	8	64	
BMI (kg/m^2^)	21.83 ± 3.69	24.96 ± 8.54	0.018
Smoke	6/54	9/95	0.95
Stroke	0/54	9/95	0.07
APTT (s)	31.76 ± 7	25.53 ± 3.41	<0.0001
PT (s)	12.14 ± 1.86	11.12 ± 0.82	<0.0001
INR	1.06 ± 0.15	0.97 ± 0.07	<0.0001
TT (s)	17.26 ± 2.08	17.69 ± 1.21	0.16
EF (%)	62.04 ± 2.73	60.59 ± 2.66	0.016
ESR (mm/h)	33.17 ± 11.91	28.86 ± 11.6	0.12
Time of surgery (min)	118.81 ± 37.19	107.77 ± 37.25	0.09
Anesthesia	6/54	3/95	0.15
VAS	3.38 (0–7)	3.18 (1–5)	0.46
Platelet (10^9^/L)	200.61 ± 69.2	146.26 ± 44.1	<0.0001
Fibrinogen (g/L)	4.44 ± 0.91	4.78 ± 0.78	0.05
D-dimer (mg/L)	2.11 ± 2.09	2.63 ± 2.12	0.25
Hypertension	4/54	35/95	0.0004
Diabetes	0/54	5/95	0.23

**Table 2:** Incidence of DVT in both groups.

Disease	DVT	Control	Total	%	*X*2	*P*
AS	8	46	54	14.8	0.89	>0.05
OA	17	78	95	17.9		

**Table 3:** Association of APTT and DVT.

	Disease	DVT	Non-DVT	*P*
APTT	AS + OA	29.95 ± 6.83	29.47 ± 5.82	0.7355
	AS	38.9 ± 13.86	29.89 ± 6.12	0.0363
	OA	28.16 ± 2.95	29.29 ± 4.97	0.3994

PT	AS + OA	11.64 ± 0.78	11.75 ± 0.83	0.6145
	AS	12 ± 0.52	11.9 ± 0.72	0.8172
	OA	11.57 ± 0.82	11.68 ± 0.87	0.6488

INR	AS + OA	1.01 ± 0.063	1.03 ± 0.071	0.4441
	AS	1.05 ± 0.049	1.04 ± 0.063	0.853
	OA	1.01 ± 0.065	1.02 ± 0.074	0.4739

**Table 4:** Association of VAS and DVT.

Disease	DVT	Non-DVT	*P*
AS + OA	3.19 (0–6)	3.16 (0–7)	0.91
AS	1.33 (0–2)	3.37 (0–7)	0.03
OA	3.12 (1–6)	3.5 (1–5)	0.24

**Table 5:** Association of D-dimer and DVT.

Disease	DVT	Non-DVT	*P*
AS + OA	3.88 ± 5.40	2.12 ± 1.97	0.01
AS	2.54 ± 0.45	0.76 ± 0.92	0.01
OA	4.05 ± 5.72	2.61 ± 2.02	0.09
